# Circular RNA PTP4A2 regulates microglial polarization through STAT3 to promote neuroinflammation in ischemic stroke

**DOI:** 10.1111/cns.14512

**Published:** 2023-10-23

**Authors:** Xingzhi Wang, Shenyang Zhang, Bingchen Lv, Hao Chen, Wei Zhang, Liguo Dong, Lei Bao, Miao Wang, Yan Wang, Wenqi Mao, Likun Cui, Ye Pang, Fei Wang, Fuling Yan, Zuohui Zhang, Guiyun Cui

**Affiliations:** ^1^ Department of Neurology The Affiliated Hospital of Xuzhou Medical University Xuzhou China; ^2^ Institute of Stroke Research Xuzhou Medical University Xuzhou China; ^3^ Jiangsu Key Laboratory of Brain Disease and Bioinformation, Research Center for Biochemistry and Molecular Biology Xuzhou Medical University Xuzhou China; ^4^ Department of Geriatrics The Affiliated Hospital of Xuzhou Medical University Xuzhou China; ^5^ Department of Neurology Affiliated to ZhongDa Hospital of Southeast University Nanjing China

**Keywords:** circPTP4A2, inflammation, ischemic stroke, microglia, STAT3

## Abstract

**Objective:**

Microglial polarization plays a critical role in neuroinflammation and may be a potential therapeutic target for ischemic stroke. This study was to explore the role and underlying molecular mechanism of Circular RNA PTP4A2 (circPTP4A2) in microglial polarization after ischemic stroke.

**Methods:**

C57BL/6J mice underwent transient middle cerebral artery occlusion (tMCAO), while primary mouse microglia and BV2 microglial cells experienced oxygen glucose deprivation/reperfusion (OGD/R) to mimic ischemic conditions. CircPTP4A2 shRNA lentivirus and Colivelin were used to knock down circPTP4A2 and upregulate signal transducer and activator of transcription 3 (STAT3) phosphorylation, respectively. Microglial polarization was assessed using immunofluorescence staining and Western blot. RNA pull‐down and RNA binding protein immunoprecipitation (RIP) were applied to detect the binding between circPTP4A2 and STAT3.

**Results:**

The levels of circPTP4A2 were significantly increased in plasma and peri‐infarct cortex in tMCAO mice. CircPTP4A2 knockdown reduced infarct volume, increased cortical cerebral blood flow (CBF), and attenuated neurological deficits. It also decreased pro‐inflammatory factors levels in peri‐infarct cortex and plasma, and increased anti‐inflammatory factors concentrations 24 h post‐stroke. In addition, circPTP4A2 knockdown suppressed M1 microglial polarization and promoted M2 microglial polarization in both tMCAO mice and OGD/R‐induced BV2 microglial cells. Moreover, circPTP4A2 knockdown inhibited the phosphorylation of STAT3 induced by oxygen–glucose deprivation. In contrast, increased phosphorylation of STAT3 partly counteracted the effects of circPTP4A2 knockdown. RNA pull‐down and RIP assays further certified the binding between circPTP4A2 and STAT3.

**Conclusion:**

These results revealed regulatory mechanisms of circPTP4A2 that stimulated neuroinflammation by driving STAT3‐dependent microglial polarization in ischemic brain injury. CircPTP4A2 knockdown reduced cerebral ischemic injury and promoted microglial M2 polarization, which could be a novel therapeutic target for ischemic stroke.

## Graphical abstract

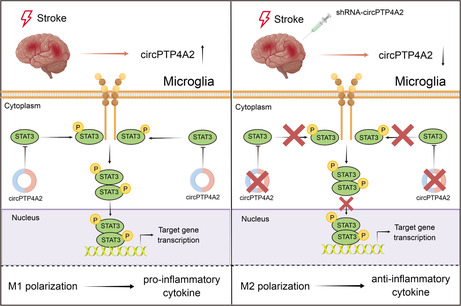

## 1 INTRODUCTION

Ischemic stroke is a prevalent cause of death and disability globally, with focal cerebral hypoperfusion caused by atherosclerotic disease or embolism being the primary mechanism underlying its pathophysiology.^1^, ^2^ Intravenous thrombolysis and endovascular thrombectomy are primary therapies for ischemic stroke, but their efficacy is highly time‐dependent and only administered to a minority of stroke patients.^3^, ^4^ Hence, there is an urgent need to explore alternative therapeutic strategies.

Neuroinflammation is a critical contributor to the pathogenesis of ischemic stroke, primarily characterized by the activation of microglia, which are innate immune cells residing within the central nervous system.^5^ Activated microglia possess two distinct polarization states, namely M1 and M2, which exert dual functions in promoting and alleviating inflammation. The M1 microglia phenotype induces neuroinflammation by expressing and releasing multiple proinflammatory mediators, such as interleukin‐6 (IL‐6), tumor necrosis factor‐α (TNF‐α), reactive oxygen species (ROS), and inducible nitric oxide synthase (iNOS), which aggravate the damage in the affected neural tissue. In contrast, the M2 microglia phenotype can phagocytose cell fragments and secrete neurotrophic and anti‐inflammatory factors, such as brain‐derived neurotrophic factor (BDNF), arginase‐1 (Arg‐1), and interleukin‐10 (IL‐10), to promote the repair of the damaged tissue.^5^, ^6^ Therefore, investigating new immunomodulatory approaches that alter the M1 phenotype toward the M2 may represent a promising strategy for preventing or reducing injury caused by ischemic stroke.

Circular RNAs (circRNAs) are non‐coding RNA molecules with a continuous loop structure formed by back‐splicing events between a downstream 5′ splice site and an upstream 3′ splice site.^7^ Mounting evidence indicates that circRNAs are implicated in ischemic stroke pathogenesis, especially via modulation of the neuroinflammatory response. For example, knocking down of circCDC14A in peripheral blood cells reduces astrocyte activation in the peri‐infarct cortex and attenuated brain damage in mice after cerebral ischemia.^8^ Moreover, circHECTD1 regulates astrocyte activation via targeting the miR142‐TIPARP pathway.^9^ Notably, there is a growing body of evidence suggesting the potential involvement of circRNAs in microglial activation in various central nervous system diseases, including spinal cord injury^10^ and epilepsy.^11^ However, it is worth mentioning that, to the best of our knowledge, there is a dearth of studies specifically investigating the role of circRNAs in stroke‐induced microglial activation and its associated processes.

Our previous report indicated that circPTP4A2 expression was elevated in patients with ischemic stroke,^12^ and this elevation of plasma circPTP4A2 was strongly associated with stroke severity.^13^ However, whether circPTP4A2 can serve as a therapeutic target in ischemic stroke and the underlying mechanisms require further investigation. Therefore, in this context, we investigated the impact of circPTP4A2 on ischemic cerebral injury using an in vivo mice model of transient middle cerebral artery occlusion (tMCAO) and both primary mouse microglia and an in vitro BV2 microglia cell system via the oxygen–glucose deprivation/reoxygenation (OGD/R) model. This study aims to explore the potential immunoregulatory mechanisms underlying the effects of circPTP4A2 on microglial polarization and establish a theoretical basis for targeting circPTP4A2 as a therapeutic intervention for ischemic stroke.

## 2 MATERIALS AND METHODS

### 2.1 Animals

C57BL/6 male (20.0–25.0 g, 7–8 weeks old) mice were purchased from the Experimental Animal Center of Xuzhou Medical University and housed under on a 12/12‐h light–dark cycle at 22°C. Water and food were available ad libitumto all mice during the study. Animal procedures were approved by the Institutional Animal Care Committee of Xuzhou Medical University and performed in accordance with Chinese Council on Animal Care Guidelines.

### 2.2 Transient middle cerebral artery occlusion (tMCAO)

The tMCAO surgery was carried out according to a previous study with minor modifications.^14^ In short, the mice were initially anesthetized with isoflurane (3%) in oxygen, and anesthesia was subsequently sustained by adjusting the isoflurane concentration as needed (1.5%). After making a midline skin incision in the neck, the proximal common carotid artery and the external carotid artery were both ligated. Then, a standardized silicone rubber‐coated 6.0 nylon monofilament (Doccol) was inserted via the right internal carotid artery and advanced to occlude the origin of the middle cerebral artery. After 60 min of occlusion in the middle cerebral artery, the blood flow was restored by removing the filament. Sham‐operated mice received identical anesthesia and surgical exposure of the arteries, but without induction of tMCAO.

### 2.3 Microinjection of circPTP4A2 shRNA lentivirus

The plasmids pFU‐GW‐016‐hU6‐CBh‐gcGFP‐IRES‐puromycin with circPTP4A2 shRNA (shRNA‐circPTP4A2) and their control (shRNA‐circCon) sequences were purchased from Genechem (Shanghai, China) and packaged into lentiviruses. The shRNA sequence for circPTP4A2 was 5′‐GTTCTAGTTTTTCGTTGGAAT‐3′. The shRNA‐circPTP4A2 or shRNA‐circCon lentivirus (2 μL of 1 × 10^9^ TU/mL) was injected into the left lateral cerebral ventricle of mice at the rate of 0.2 μL/min. The coordinates for the lateral ventricle were: −0.3 mm anteroposterior, 1.0 mm lateral and 2.2 mm ventral. Two weeks after lentivirus injections, the mice underwent MCAO surgery as described above.

### 2.4 2,3,5‐Triphenyl tetrazolium chloride (TTC) staining

After being anesthetized with 1% pentobarbital sodium, the brains of mice were rapidly removed at 3 days after tMCAO. The fresh brains were kept at a temperature of −20°C for 30 min before being rapidly sliced into 2 mm coronal sections. Afterward, these sections were immersed in a 2% TTC solution in phosphate buffer and stained in the dark at 37°C for 20 min. Subsequently, the sections were incubated overnight at 4°C in a 4% paraformaldehyde (PFA) solution for fixation. Following TTC staining, digital camera‐mediated imaging was conducted on the sections to measure the infarct areas of each section through the utilization of Image J software. The total infarct volume was computed according to the following equation: (contralateral hemisphere volume – non‐infarcted ipsilateral hemisphere volume)/contralateral hemisphere volume × 100%.

### 2.5 Cerebral blood flow (CBF) measurements

The laser speckle imaging system (RWD, Shenzhen, China) was used to measure the total blood flow in the cortex according to manufacturer's instructions. In short, charge‐coupled device image sensor was positioned above the anesthetized mouse's head and a 785 nm laser diode was used to illuminate the intact skull surface, allowing for diffuse penetration of laser through the brain. The CBF was assessed bilaterally and recorded 15 min prior to the induction of MCAO, throughout the period of ischemia, and up to 15 min after reperfusion onset. The experimental mice were shielded from direct exposure to sunlight and infrared radiation, while the ambient temperature of the room was maintained at 26°C. To assess changes in CBF, we defined a region of interest (ROI) that encompassed the right cortical infarct area located posterior to the coronal suture and medial to the linear temporalis. Experimental mice that did not exhibit a reduction in CBF of at least 75% compared to baseline or experienced mortality rates below 10% following ischemic induction were excluded from the study due to the high probability (>95%) of infarction when early CBF drops below 25% of the control.^15^, ^16^

### 2.6 Behavioral tests

Prior to tMCAO surgery, all mice underwent five consecutive days of twice daily training to minimize anxiety and ensure equivalent baseline levels in both control and experimental groups. The neurological function of mice was evaluated using modified neurological severity score (mNSS) assessment, foot fault test and adhesive removal test prior to tMCAO surgery as well as at 1, 3, 5, and 7 days post‐surgery.

The mNSS consists of several tests, including motor testing, timed beam balance test, sensory (visual and tactile) tests, reflex test to sudden auditory stimuli, and corneal reflex test. The neurological function is graded on a scale from 0 to 14, with the normal score being 0 and the maximal deficit score being 14.^17^ The foot fault test was conducted to evaluate sensorimotor deficits. During the test, individual mice were placed on an elevated 10‐mm square wire mesh with a total grid area of 40 cm × 40 cm. The mice were allowed to walk freely for 2 min while being videotaped, and both the number of foot faults and the total number of steps taken were documented. The percentage of foot faults was then calculated as the ratio of the number of foot faults to the total number of steps taken.^18^ An adhesive removal test was conducted to quantify somatosensory deficits, following established protocols.^19^ Briefly, two small adhesive tape pieces measuring 4 × 3 mm were carefully attached to the distal radial region of each forelimb in an alternating sequence, with consistent pressure applied by the experimenter before each trial. Subsequently, the animals were released into a testing cage, and the precise timing of both initial contact and subsequent removal of the adhesive patch was recorded. Contact was considered to have occurred when either paw shaking or mouth contact was observed. The trial concluded upon the removal of the adhesive patch or after a duration of 2 min had elapsed. Prior to the surgical intervention, the animals underwent a comprehensive preoperative training regimen, consisting of twice‐daily sessions over a period of 3 days. Following the surgery, postoperative assessments were conducted on days 1, 3, 5, and 7.

### 2.7 Cell culture, lentiviral transduction, and OGD/R treatment

In this study, primary cultured mouse microglia were obtained from postnatal C57BL/6J mice (P1–P2). The brain tissue was carefully dissected to remove the meninges and blood vessels, followed by enzymatic digestion using trypsin–EDTA. The dissociated cells were plated onto poly‐L‐lysine‐coated cell culture flasks containing Dulbecco's modified Eagle's medium (DMEM) supplemented with 10% fetal bovine serum (FBS) and 1% penicillin–streptomycin. After 7 days, 0.25 ng/mL of colony‐stimulating factor 2 [granulocyte‐macrophage] (CSF2/GM‐CSF) was added to the flasks to promote microglial proliferation. The microglia were subsequently detached from the flasks by gentle shaking and collected from the cell medium through centrifugation at 1500 *g* for 5 min.

The BV2 microglial cell line used in this study was from our laboratory's stock. The BV2 cells were cultured in 6‐well plates with Dulbecco's modified Eagle's medium (DMEM) / high glucose supplemented with 10% fetal bovine serum, 120 U/mL penicillin, and 100 mg/L streptomycin, and were then incubated in a 37°C CO_2_ incubator. Lentiviral vectors (Genechem) carrying circControl shRNA or circPTP4A2 shRNA were used to transduce BV2 cells with a multiplicity of infection of 10. Furthermore, to investigate the regulatory role of circPTP4A2 in the STAT3 activation, BV2 cells were incubated with a STAT3 activator Colivelin (0.5 μM, MCE, USA).^20^

Following a 48 h transduction with lentivirus, microglial cells were exposed to 3 h of OGD in vitro to simulate ischemia. In brief, the culture medium was substituted with the glucose‐free medium. Subsequently, the cells were placed in a humidified 37°C incubator with a gas mixture consisting of 1% O_2_, 5% CO_2_, and 94% N_2_ for a period of 3 h. After that, the cells were transferred to normal culture medium for 24 h and maintained at 37°C in an incubator with 5% CO_2_ for reperfusion.

### 2.8 RNA isolation and quantitative polymerase chain reaction (qPCR)

Peri‐infarct cortex, plasma, and white blood cells (WBCs) were isolated and collected from mice after 24 h of tMCAO‐induced ischemia. Primary mouse microglia or BV2 cells were harvested immediately after inducing OGD/R. The total RNA from tissue samples and cultured cells were extracted according to the manufacturer's protocol, which utilized the Trizol method (Invitrogen) and miRNeasy Mini kit (Qiagen). The concentration of RNA was determined with a NanoPhotometer® spectrophotometer (IMPLEN, USA). For the qPCR assay, cDNA was synthesized using the HiScript® Q RT SuperMix for qPCR Kit (Vazyme, China), and subsequently, the reactions were performed in the Bio‐Rad CFX96 Real‐Time system utilizing the UltraSYBR Mixture (CWBIO, China). The cycling conditions for performing qPCR were carried out as per the manufacturer's recommended protocol, with each qPCR assay being performed in triplicate. The relative gene expression was analyzed by the 2^−ΔΔCt^ method and normalized to GAPDH (glyceraldehyde‐3‐phosphate dehydrogenase). The primer sequences were designed and synthesized by RiBoBio (China) as described in Table S1.

### 2.9 Cytokine and chemokine measurements

Following tMCAO procedure, blood (≈500 μL) and peri‐infarct cortex were collected from mice after 24 h of ischemia. Ice‐cold phosphate‐buffered solution (PBS) was then used to prepare approximately 200 μL of plasma and 10% (wt/vol) brain homogenates. ELISA Kits (Abcam) were used to measure the levels of TNF‐α, IL‐1β, IL‐10, and TGF‐β1, and the procedures followed were in accordance with the manufacturer's instructions.

### 2.10 Immunofluorescence

Brain cryosections or treated BV2 cells were fixed with 4% paraformaldehyde (PFA) for 20 min, followed by permeabilization using a mixture of 0.5% Triton X‐100, 10% donkey serum, and 90% PBS for 60 min at room temperature. The samples underwent overnight incubation at 4°C with the following primary antibodies: anti‐Iba‐1 (1:500, rabbit, 019–19,741, WAKO, Japan), anti‐CD16 (1:500, goat, PA5‐47230, Thermo Fisher Scientific, USA), and anti‐CD206 (1:500, rat, MA5‐16871, Thermo Fisher Scientific, USA). After being washed with PBS, the brain slices/cells were incubated with secondary antibodies conjugated to either Alexa Fluor 488 or Alexa Fluor 546 (1:500, Vector Laboratories, USA) at room temperature for 60 min. Subsequently, the samples were washed three times with PBS, and then stained with 100 nM DAPI for 15 min. Immunofluorescent staining was observed and photographed using a fluorescence microscope (Olympus Corporation).

### 2.11 Western blotting

The expression of proteins from peri‐infarct cortex and OGD/R‐induced BV2 cells was analyzed by Western blot. In short, brain/cells samples were lysed with RIPA buffer (Beyotime Biotechnology, China) for 10 min, followed by centrifugation at 13,000 *g* for 30 min at 4°C. Subsequently, the protein extracts were subjected to boiling for 5 min, separated through 10% SDS‐PAGE gel electrophoresis, and eventually transferred onto nitrocellulose (NC) membranes (Amersham Biosciences, Germany). The NC membranes were blocked using 5% non‐fat milk and then incubated overnight at 4°C with the following primary antibodies: anti‐Iba‐1 (1:1000, rabbit, 17,198, CST, USA), anti‐CD16 (1:500, rabbit, MA5‐36143, Thermo Fisher Scientific, USA), anti‐CD11b (1:1000, rabbit, 17,800, CST, USA), anti‐CD206 (1:500, rabbit, ab64693, Abcam, UK), anti‐Arginase‐1 (1:1000, rabbit, 89,872, CST, USA), anti‐phospho‐STAT3 (1:1000, rabbit, 9145, CST, USA), anti‐STAT3 (1:1000, rabbit, 30,835, CST, USA), anti‐GAPDH (1:1000, mouse, 60,004‐1‐Ig, Protein Tech, USA), and anti‐Histone H3 (1:1000, mouse, 14,269, CST, USA). The next day, the NC membrane was washed in TBST before being incubated at room temperature for 60 min with the HRP‐conjugated secondary antibodies (1:1000, Protein Tech, USA). Bands were visualized using WesternLumaxLight SuperiorHRP substrate reagent (ZETA‐Life, USA) with a gel imaging system (ChemiDoc XRS+, Bio‐Rad, USA). The quantification of individual protein bands was performed using densitometry analysis with the ImageJ software.

### 2.12 Fluorescence in situ hybridization (FISH)

The FISH assay was performed using the RNA FISH Kit (Gene‐Pharma, China) according to the manufacturer's instructions. In brief, BV2 cells were seeded in confocal dishes and fixed with 4% PFA, and permeabilized with 0.25% Triton X‐100 in PBS for 15 min. Subsequently, the cells were incubated with 100 μL FAM‐labeled circPTP4A2 probe in a hybridization buffer at 73°C for 5 min, followed by an overnight hybridization reaction at 37°C. Following the washing and blocking steps, the samples were subjected to an overnight incubation with anti‐STAT3 antibody (1:500, rabbit, 30,835, CST, USA) at 4°C on the second day of the experiment. Finally, cell nuclei were stained with DAPI and analyzed using a confocal microscope (Leica, Germany). The circPTP4A2 probe, labeled with FAM at the 5′ end, was designed and synthesized by Gene‐Pharma (China) with the specific sequence 5′‐ CGTATATTCCAACGAAAAACTAGAACGTGGATTCCTTCTT ‐3′.

### 2.13 Nuclear and cytoplasmic isolation

The nuclear and cytoplasmic fractions of BV2 cells were isolated using Paris Kit (Ambion, USA) and the RNA was then extracted for further analysis via qPCR. The positive controls for cytoplasmic and nuclear RNA were GAPDH and U6, respectively.

### 2.14 RNA pull‐down

RNA pull‐down assay was conducted using a Pierce Magnetic RNA‐Protein Pull‐Down Kit (Thermo Fisher Scientific, USA). Biotinylated probes for circPTP4A2 and control were designed and synthesized by RiboBio (China) with their corresponding sequences provided in Table S2. Lysis and 4 h incubation of approximately 1 × 10^7^ BV2 cells with biotinylated probes at 4°C was followed by incubation with 50 μL of streptavidin‐coated magnetic beads at room temperature. The retrieved bead‐probe‐protein complex was washed and boiled in SDS buffer before being separated using SDS‐PAGE. Finally, the separated proteins were detected using an antibody specific to STAT3 (CST, USA) to determine expression levels.

### 2.15 RNA immunoprecipitation (RIP)

RIP was carried out using Magna RIP RNA‐Binding Protein Immunoprecipitation Kit (Millipore, USA) as per the manufacturer's instructions. In brief, BV2 cells (2 × 10^7^) were trypsinized, washed twice with ice‐cold PBS, and then resuspended in an equal pellet volume of RIP lysis buffer containing a protease inhibitor cocktail and RNase inhibitor. Next, 5 μg of anti‐STAT3 antibody (CST, USA) was added to magnetic beads, and the mixture was agitated and incubated for 30 min at room temperature in RIP wash buffer. Subsequently, the magnetic beads were washed thrice with RIP wash buffer, followed by overnight incubation with cell lysates at 4°C. After that, the immunoprecipitates were suspended in proteinase K buffer (1.2 μg/μL proteinase K and 1% SDS) and incubated at 55°C for 30 min. CircPTP4A2 was then extracted using phenol, chloroform, and isoamyl alcohol according to the manufacturer's instructions and subsequently subjected to qPCR analysis.

### 2.16 Statistical analysis

All statistical analyses were performed using GraphPad Prism 8.0 (GraphPad Software, USA) or SPSS 26.0 (IBM Corporation, USA). The data were presented as mean ± standard deviation (SD). Normality of data distribution was assured using Shapiro–Wilk test. For the comparison of differences between two groups, normally distributed continuous variables were assessed using the Student's *t* test, whereas non‐normally distributed variables were evaluated using the Mann–Whitney *U*‐test. In the case of multiple comparisons involving more than two groups, the data were subjected to a one‐way analysis of variance (ANOVA). Subsequently, for normally distributed variables, post‐hoc Holm–Sidak tests were performed. Conversely, for non‐normally distributed variables, the Kruskal–Wallis test was employed. Correlations were assessed by computing Pearson's correlation coefficients. A two‐sided *p* < 0.05 was considered to indicate a statistically significant difference.

## 3 RESULTS

### 3.1 Gene information and expression of circPTP4A2 in plasma, WBC and peri‐infarct cortex of tMCAO mice

**FIGURE 1:**
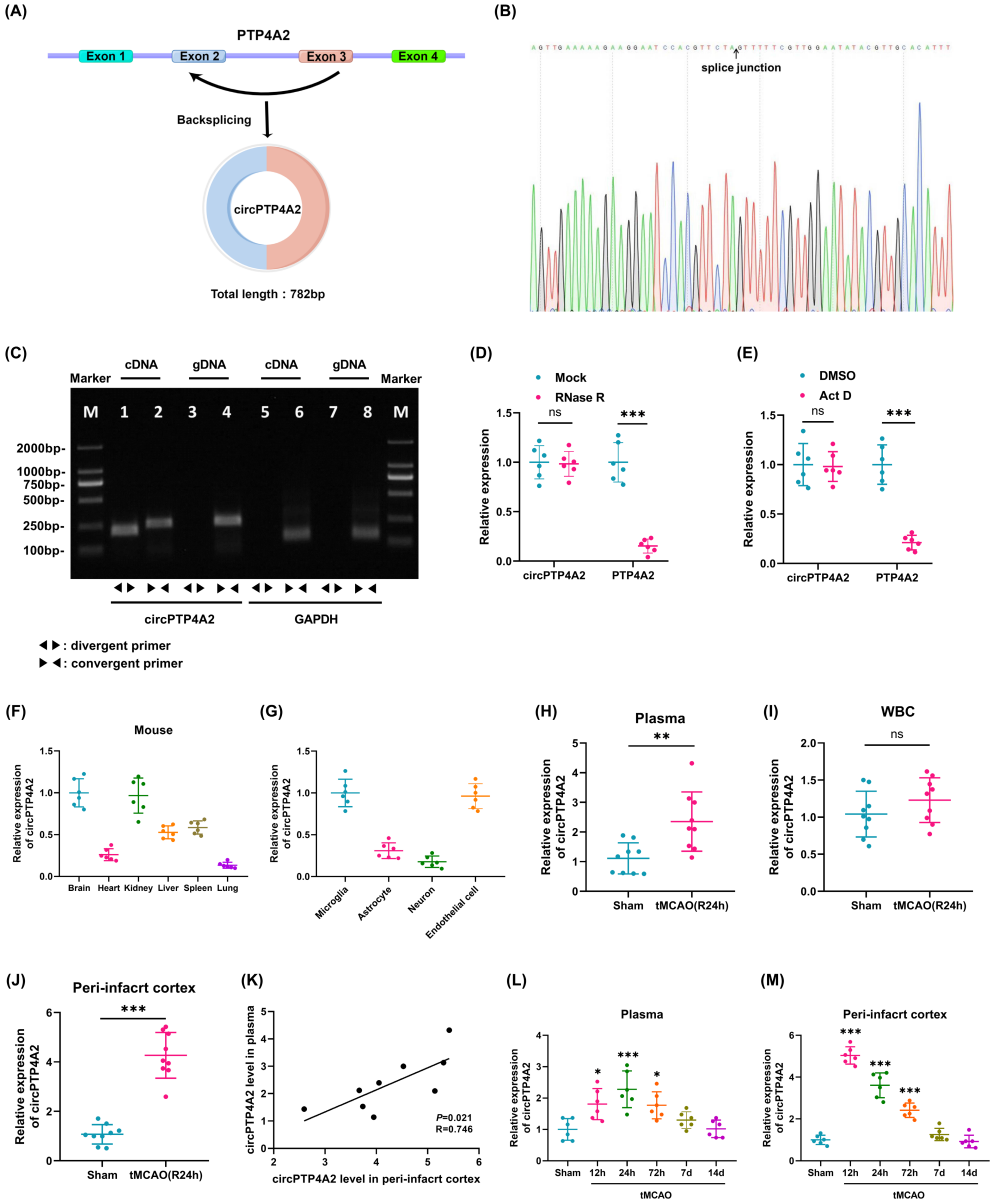
Gene information and the expression of circPTP4A2 in tMCAO mice. (A) Schematic illustration showing the circularization of PTP4A2 exon 2–3 forming circPTP4A2. (B) Confirmation of the back‐splicing junction site within the circPTP4A2 by Sanger sequencing. The red arrow indicates the specific splicing junction of circPTP4A2. (C) RT‐PCR for the analysis of the existence of circPTP4A2 in brain tissue. Divergent primers amplified circPTP4A2 in cDNA but not genomic DNA (gDNA). GAPDH was used as negative control. (D) The expression levels of circPTP4A2 and PTP4A2 mRNA were determined by qPCR in brain tissue treated with or without RNase R. The relative levels of circPTP4A2 and PTP4A2 mRNA were normalized to the values measured in the mock treatment group. *n* = 6. ***p < 0.001; ns, not significant. Student's *t* test. (E) The expression levels of circPTP4A2 and PTP4A2 mRNA were determined by qPCR in brain tissue treated with or without Actinomycin D (Act D). The relative levels of circPTP4A2 and PTP4A2 mRNA were normalized to the values measured in the DMSO treatment group. *n* = 6. ***p < 0.001; ns, not significant. Student's *t* test. (F) Relative expression of circPTP4A2 was determined by qPCR in brain, heart, kidney, liver, spleen, and lung of mice. *n* = 6. (G) Relative expression of circPTP4A2 was determined by qPCR in primary cultured microglia, astrocytes, neurons, and endothelial cells. (H–J) The expression of circPTP4A2 in plasma (H), WBC (I), and peri‐infarct cortex (J) after tMCAO. *n* = 9. **p < 0.01; ***p < 0.001; ns, not significant. Student's *t* test. (K) The correlation between plasma circPTP4A2 levels and brain tissue peri‐infarct cortex after tMCAO. *n* = 9. (L) Temporal changes in plasma circPTP4A2 levels during the post‐stroke period of 12 h to 14 days. *n* = 6. *p < 0.05, ***p < 0.001 versus Sham group. (M) Temporal changes in peri‐infarct cortex circPTP4A2 levels during the post‐stroke period of 12 h to 14 days. *n* = 6. ***p < 0.001 versus Sham group.

According to the circBase database^21^ and NCBI reference sequence, circPTP4A2 is generated through the back‐splicing of exon 2–3 within the protein tyrosine phosphatase 4A2 (PTP4A2) gene (Figure 1A) and demonstrates high species conservation. The 5′ to 3′ exon junction within circPTP4A2 was localized using Sanger sequencing (Figure 1B). Convergent and divergent primers were designed to amplify PTP4A2 mRNA and circPTP4A2, respectively. The RT‐PCR indicated that circPTP4A2 was specifically amplified by divergent primers in cDNA, whereas no detectable amplification was observed in gDNA (genomic DNA; Figure 1C). Additionally, qPCR confirmed that circPTP4A2 was resistant to RNase R, while PTP4A2 mRNA was significantly reduced following RNase R treatment (Figure 1D). After a 24‐h treatment with actinomycin D, transcriptional inhibition resulted in a significant decrease in PTP4A2 expression levels, while the expression of circPTP4A2 remained unaffected (Figure 1E). Furthermore, circPTP4A2 exhibited relatively higher expression levels in the brain and kidney of mice as compared to heart, liver, spleen, and lung (Figure 1F). Notably, circPTP4A2 showed prominent expression in microglia and endothelial cells when examined in primary cultures of microglia, astrocytes, neurons, and endothelial cells (Figure 1G).

To test the involvement of circPTP4A2 in experimental stroke models, plasma, WBC, and peri‐infarct cortex were collected at 24 h after tMCAO. qPCR demonstrated that circPTP4A2 levels were significantly increased in plasma (Figure 1H; *p* < 0.01) and peri‐infarct cortex (Figure 1J; *p* < 0.001) after tMCAO, and the increase in peri‐infarct cortex were more evident. Although there was a trend toward elevated circPTP4A2 levels in WBC after tMCAO, this did not meet statistical significance when comparing circPTP4A2 levels in sham‐operated mice (Figure 1I). In addition, the correlation analysis demonstrated a positive correlation between plasma circPTP4A2 levels and peri‐infarct cortex circPTP4A2 expression levels (Figure 1K; *p* < 0.05).

Subsequently, the expression levels of circPTP4A2 in both plasma and peri‐infarct cortex of tMCAO mice were monitored for a period of 14 days post‐tMCAO. In plasma, circPTP4A2 exhibited an initial upward trend within 12 h and a significant increase by 24 h after reperfusion. This significant elevation in circPTP4A2 levels persisted until 72 h and returned to baseline levels by the 7th day after tMCAO (Figure 1L). Conversely, in the peri‐infarct cortex, circPTP4A2 reached its peak level at 12 h after tMCAO modeling and subsequently returned to normal levels by the 7th day after tMCAO (Figure 1M). The presence of this temporal discrepancy suggests that the increase in circPTP4A2 in the peri‐infarct cortex preceded that observed in the plasma.

### 3.2 Knockdown of circPTP4A2 expression decreased cerebral injury and pro‐inflammatory cytokine levels

**FIGURE 2:**
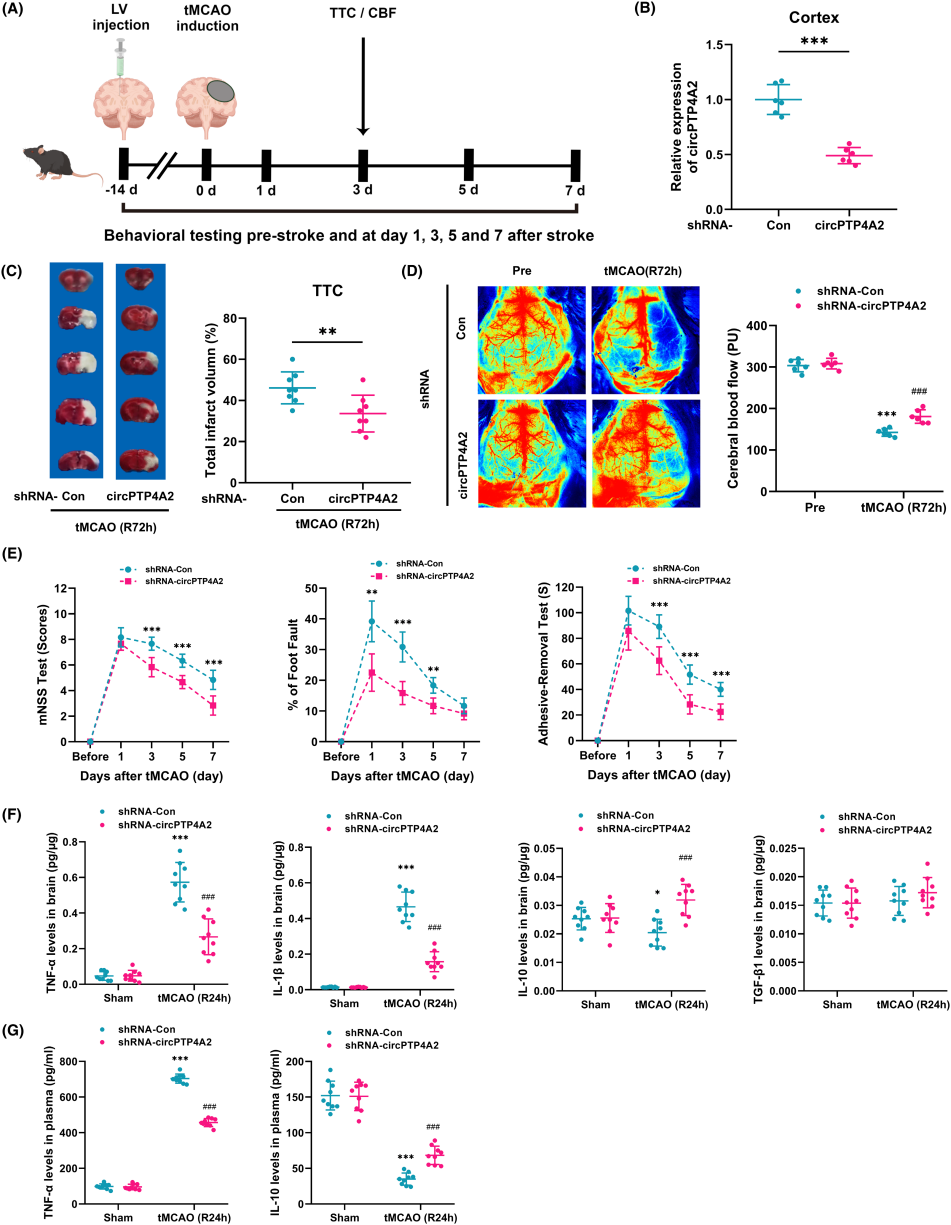
Effects of circPTP4A2 on cerebral injury and inflammatory cytokines in tMCAO mice. (A) Flow chart of the experiment design. (B) Relative expression of circPTP4A2 in peri‐infarct cortex 14 days after circPTP4A2 shRNA lentivirus microinjection, as determined by qPCR. *n* = 6. **p < 0.001. Student's *t* test. (C) Brain infarct volume at 72 h after tMCAO was analyzed by TTC staining. *n* = 8. **p < 0.01. Student's *t* test. (D) Representative images of laser speckle imaging system and quantitative analyses of cortical CBF before tMCAO (Pre) or 72 h after tMCAO. *n* = 6. ***p < 0.001 versus the Pre + shRNA‐Con group. ^###^ *p* < 0.001 versus the tMCAO + shRNA‐Con group. Two‐way ANOVA followed by Holm–Sidak post hoc multiple‐comparison tests. (E) Neurological deficits were evaluated by the mNSS test, foot fault assays, and adhesive removal test in 1, 3, 5, and 7 days after tMCAO. *n* = 6. **p < 0.01; ***p < 0.001 versus the tMCAO + shRNA‐Con group. Student's *t* test. (F,G) Effects of intraventricular injection of circPTP4A2 shRNA and subsequent knockdown of circPTP4A2 on the levels of TNF‐α, IL‐1β, IL‐10 and TGF‐β1 in peri‐infarct cortex, as well as the levels of TNF‐α and IL‐10 in plasma by ELISA at 24 h after tMCAO. *n* = 9. *p < 0.05; ***p < 0.001 versus the Sham + shRNA‐Con group. ^###^ *p* < 0.001 versus the tMCAO + shRNA‐Con group. Two‐way ANOVA followed by Holm–Sidak post hoc multiple‐comparison tests.

To assess the role of circPTP4A2 in the pathogenesis of stroke in vivo, we performed a lateral ventricle microinjection of lentivirus 2 weeks before tMCAO modeling with the aim of downregulating the expression of circPTP4A2 in the brain cortex (Figure 2A). Two weeks following the lentivirus microinjections, qPCR analysis revealed a significant decrease in circPTP4A2 expression in mice that received circPTP4A2 shRNA compared to those that received Control shRNA, with a knockdown efficiency of up to 50% (Figure 2B). The infarct volume, as evaluated using TTC staining, was significantly decreased at 72 h after tMCAO in the circPTP4A2 shRNA‐injected mice compared with the Control shRNA‐injected mice (Figure 2C). In addition, the lentiviral treatment of circPTP4A2 shRNA significantly increased cortical CBF at 72 h after tMCAO compared to the lentiviral treatment of Control shRNA (Figure 2D). Besides, the neurological function of mice was evaluated using the mNSS test, foot fault test, and adhesive‐removal test at 1, 3, 5, and 7 days after tMCAO. Our results also indicated that microinjection of circPTP4A2 shRNA lentivirus significantly ameliorated neurological deficits (Figure 2E).

Subsequently, we investigated whether the altered expression of circPTP4A2 could modulate the inflammatory response after tMCAO. The levels of two pro‐inflammatory cytokines (TNF‐α and IL‐1β) and two anti‐inflammatory cytokines (IL‐10 and TGF‐β1) were measured in either the peri‐infarct cortex or plasma of mice at 24 h after tMCAO via ELISA assay. Compared with the Control shRNA lentivirus treatment, administration of circPTP4A2 shRNA lentivirus reduced TNF‐α and IL‐1β levels induced by tMCAO and facilitated elevations in IL‐10 levels in the peri‐infarct cortex (Figure 2F). Similarly, the circPTP4A2 shRNA lentivirus treatment led to a significant decrease in tMCAO‐induced TNF‐α levels and an increase in IL‐10 levels in plasma compared to the Control shRNA lentivirus treatment (Figure 2G). These results suggest a pivotal role of circPTP4A2 in modulating the immune response following ischemic stroke.

### 3.3 Knockdown of circPTP4A2 expression facilitated M2 microglia polarization in vivo

**FIGURE 3:**
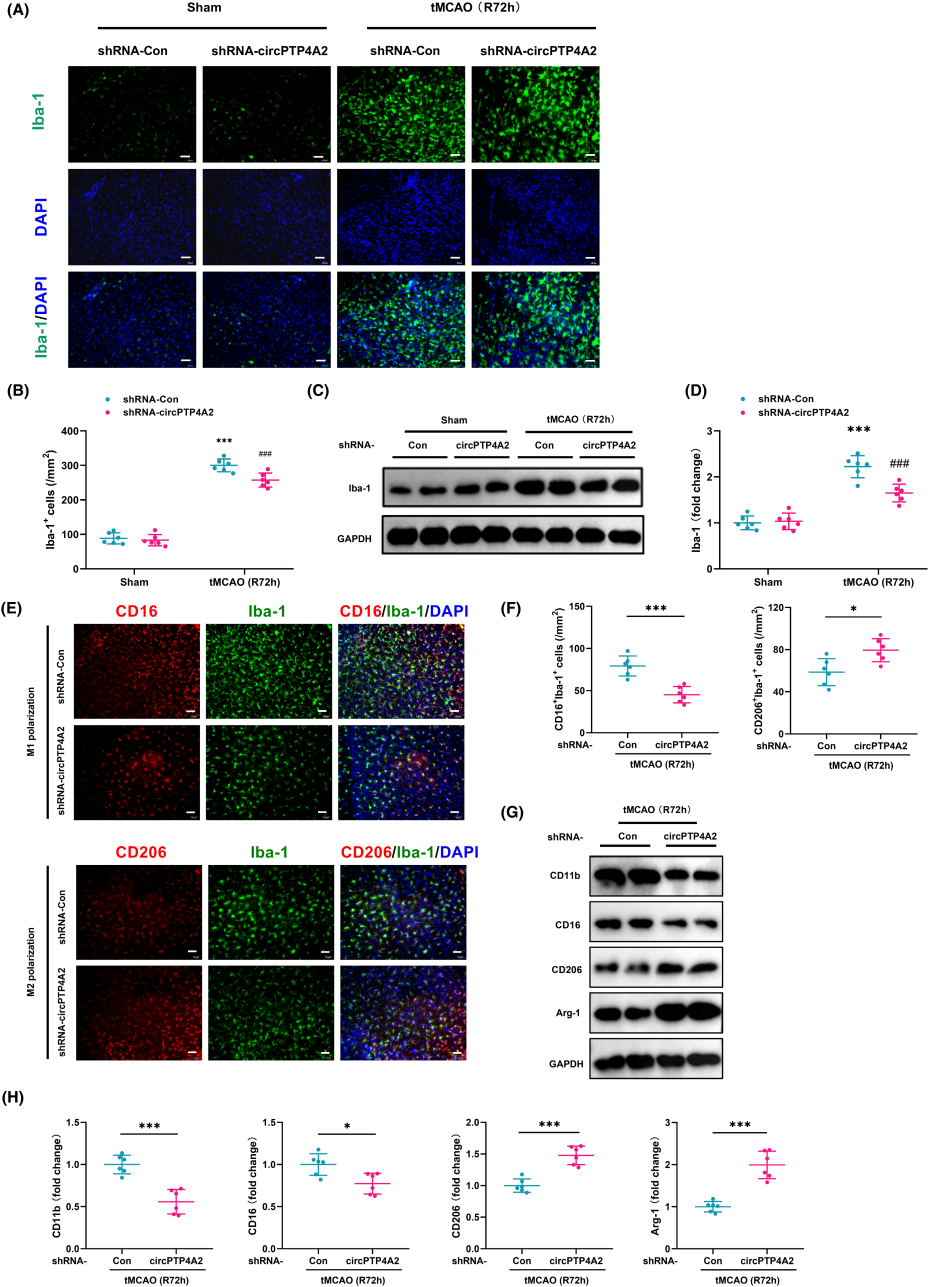
Knockdown of circPTP4A2 modulated microglial polarization in vivo. (A) Representative images of microglia immunostained for Iba‐1 in peri‐infarct cortex at 72 h after tMCAO. Green, Iba‐1; Blue, DAPI. Scale bar: 50 μm. (B) Quantification of the number of Iba‐1 positive cells using ImageJ software. *n* = 6. ***p < 0.001 versus the Sham + shRNA‐Con group. ^###^ *p* < 0.001 versus the tMCAO + shRNA‐Con group. Two‐way ANOVA followed by Holm–Sidak post hoc multiple comparison tests. (C,D) Western blot analysis of Iba‐1 in peri‐infarct cortex at 72 h after tMCAO. *n* = 6. ***p < 0.001 versus the Sham + shRNA‐Con group. ^###^ *p* < 0.001 versus the tMCAO + shRNA‐Con group. Two‐way ANOVA followed by Holm–Sidak post hoc multiple comparison tests. (E) Representative images of double immunostaining of microglial polarization in peri‐infarct cortex at 72 h after tMCAO. M1 polarization: CD16^+^ (red) and Iba‐1^+^ (green); M2 polarization: CD206^+^ (red) and Iba‐1^+^ (green). Scale bar: 50 μm. (F) Quantification of the number of CD16^+^Iba‐1^+^ and CD206^+^Iba‐1^+^ positive cells using ImageJ software. *n* = 6. *p < 0.05; ***p < 0.001. Student's *t* test. (G,H) Western blot analysis of CD11b, CD16, CD206, and Arg‐1 in peri‐infarct cortex at 72 h after tMCAO. *n* = 6. *p < 0.05; ***p < 0.001. Student's *t* test.

Considering the critical role of microglia in stroke‐induced neuroinflammation, we further investigated the impact of circPTP4A2 on microglial polarization after tMCAO. We firstly performed immunolabeling on brain sections using Iba‐1 as a microglial surface marker, and quantified the number of Iba‐1 positive cells in the peri‐infarct cortex. We found a significant increase in Iba‐1 expression in the peri‐infarct cortex of the tMCAO group compared to the sham group. Furthermore, at 72 h after tMCAO, the number of Iba1‐labeled microglia in the peri‐infarct cortex was significantly reduced in the circPTP4A2 shRNA group compared to the Control shRNA group (Figure 3A,B). To further confirm this finding, we performed Western blot analysis to determine the expression of Iba‐1 in the peri‐infarct cortex. The results showed that 72 h after tMCAO, the level of Iba‐1 expression was significantly lower in the group treated with circPTP4A2 shRNA compared to the Control shRNA‐treated group (Figure 3C,D).

To further investigate whether circPTP4A2 affects microglial polarization phenotype, we assessed the microglial polarization state using surface markers specific for M1 and M2. Compared to treatment with Control shRNA lentivirus, the lentiviral treatment using circPTP4A2 shRNA significantly reduced the number of M1 microglia (CD16^+^Iba‐1^+^) in the peri‐infarct cortex at 72 h after tMCAO, while also increasing the number of M2 microglia (CD206^+^Iba‐1^+^), indicating a transition from M1 to M2 microglial phenotype (Figure 3E,F). The immunofluorescence microscopy findings were subsequently validated by western blotting. Specifically, the protein levels of CD11b and CD16, which are markers of M1 microglia, were observed to decrease, whereas those of CD206 and Arg‐1, markers of M2 microglia, were found to increase in tMCAO‐treated mice after circPTP4A2 knockdown (Figure 3G,H). These results indicate that the knockdown of circPTP4A2 can reduce microglial activation induced by tMCAO while facilitating M1–M2 phenotypic polarization.

### 3.4 Knockdown of circPTP4A2 expression facilitated M2 microglia polarization in vitro

**FIGURE 4:**
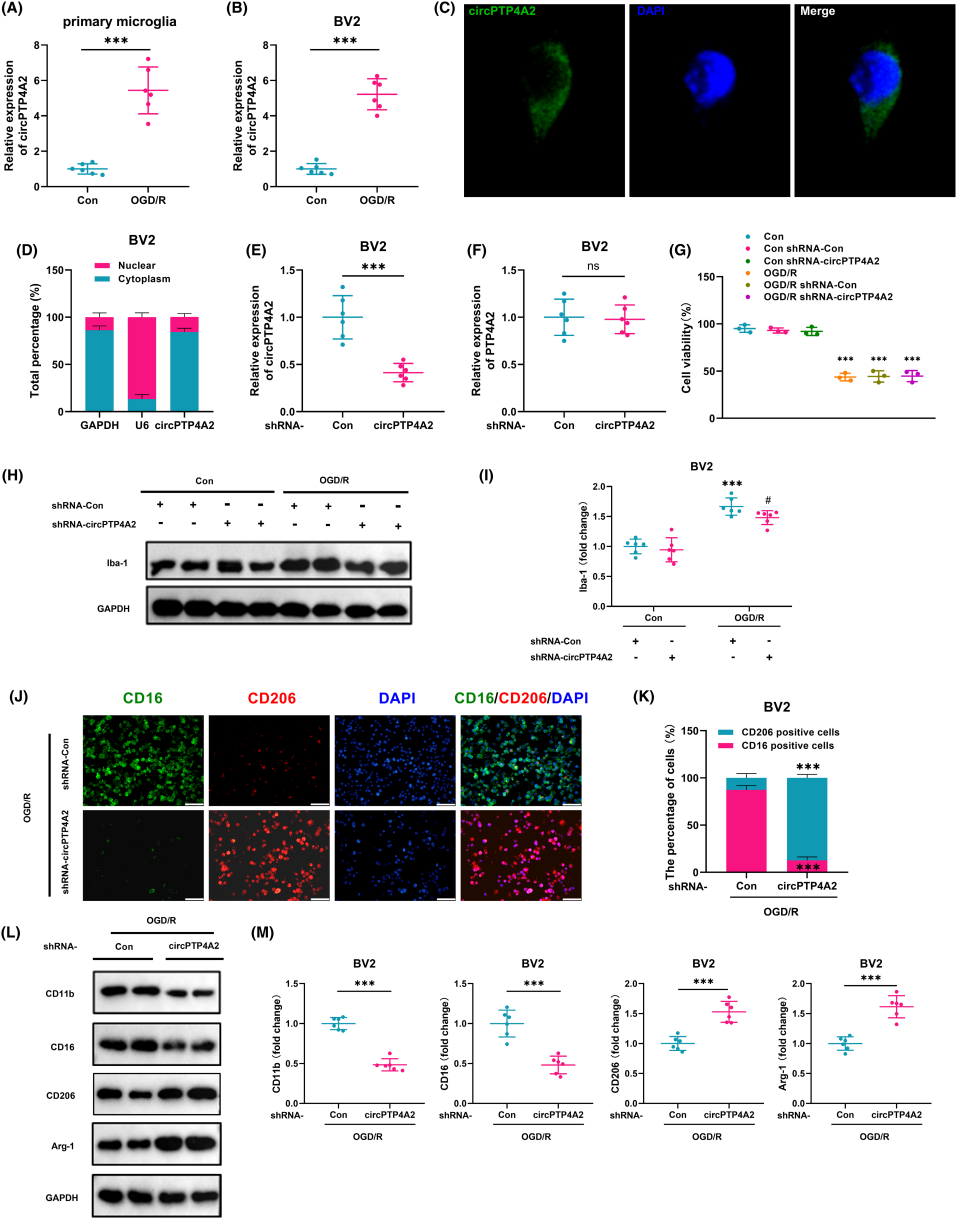
Knockdown of circPTP4A2 modulated microglial polarization in vitro. (A,B) Relative expression of circPTP4A2 in primary mouse microglia (A) and BV2 cells (B) after OGD/R. Cells were treated with OGD for 3 h and reperfusion for 24 h. *n* = 6. ***p < 0.001. Student's *t* test. (C) The localization of circPTP4A2 in BV2 cells was assessed by FISH. (D) circPTP4A2 expression in the cytoplasm and nucleus of BV2 cells was detected by qPCR. GAPDH, cytoplasmic control; U6, nuclear control. (E,F) Effects of circPTP4A2 and PTP4A2 expression in BV2 cells after circPTP4A2 shRNA lentivirus transduction, as determined by qPCR. Cells were transduced with circPTP4A2 shRNA lentivirus for 48 h and then the expression levels of circPTP4A2 and PTP4A2 were measured. *n* = 6. ***p < 0.001; ns, not significant. Student's *t* test. (G) Cell viability assessed by CCK‐8 assay. *n* = 6. ***p < 0.001 versus the control group. One‐way ANOVA followed by the Holm–Sidak test. (H,I) Western blot analysis of Iba‐1 in BV2 cells. Cells were transduced with circPTP4A2 shRNA lentivirus for 48 h and treated with OGD for 3 h and reperfusion for 24 h. *n* = 6. ***p < 0.001 versus the control shRNA‐Con group. ^#^ *p* < 0.05 versus the OGD/R‐treated shRNA‐Con group. (J) Representative images of CD206 and CD11b double immunostaining in BV2 cells treated with OGD/R. (K) Analysis of the percentage of CD16‐ or CD206‐positive cells using ImageJ software. *n* = 6. ***p < 0.001, Student's *t* test. (L,M) Western blot analysis of CD11b, CD16, CD206, and Arg‐1 in BV2 cells treated with OGD/R. *n* = 6. ***p < 0.001. Student's *t* test.

As our in vivo study indicated the involvement of circPTP4A2 in microglia polarization, we further aimed to explore the role of circPTP4A2 in vitro for microglia polarization. As shown in Figure 4A,B, the expression of circPTP4A2 was significantly increased in primary mouse microglia and BV2 cells treated with OGD/R. We next investigated the intracellular distribution of circPTP4A2 in vitro and observed that, similar to most circRNAs, circPTP4A2 was primarily distributed in the cytoplasm of BV2 cells, which was confirmed by FISH (Figure 4C) and qPCR (Figure 4D). Transducing BV2 cells with lentivirus carrying circPTP4A2 shRNA led to a reduction in the expression of circPTP4A2, with no changes detected in PTP4A2 mRNA expression (Figure 4E,F). The CCK8 assay results demonstrated that OGD/R treatment significantly decreased the viability of BV2 cells. However, the lentiviral transfection of circPTP4A2 shRNA did not have a significant effect on the viability of BV2 cells induced by OGD/R (Figure 4G).

Immunofluorescent staining revealed that BV2 microglial cells labeled with Iba‐1 displayed a highly ramified morphology in the resting state, which transformed into an activated ameboid morphology characterized by an increase in cell body size and shorter, thicker ramifications after 24 h of exposure to OGD/R. However, the lentiviral transfection of circPTP4A2 shRNA had no effect on morphology of BV2 microglia cells (Figure S1). Moreover, transfection of BV2 cells with the circPTP4A2 shRNA lentivirus resulted in a decrease in the percentage of CD16‐labeled M1 microglia and an increase in the percentage of CD206‐labeled M2 microglia under conditions of OGD/R induction (Figure 4J,K). Western blot analysis also revealed that knockdown of circPTP4A2 partially reversed the OGD/R induced upregulation of CD11b and CD16, as well as the downregulation of CD206 and Arg1 (Figure 4L,M). Our data demonstrate that knockdown of circPTP4A2 impairs the activation of BV2 microglia induced by OGD/R and promotes polarization from M1 to M2 phenotype.

### 3.5 circPTP4A2 knockdown promotes microglial M1 to M2 polarization via downregulation of STAT3

**FIGURE 5:**
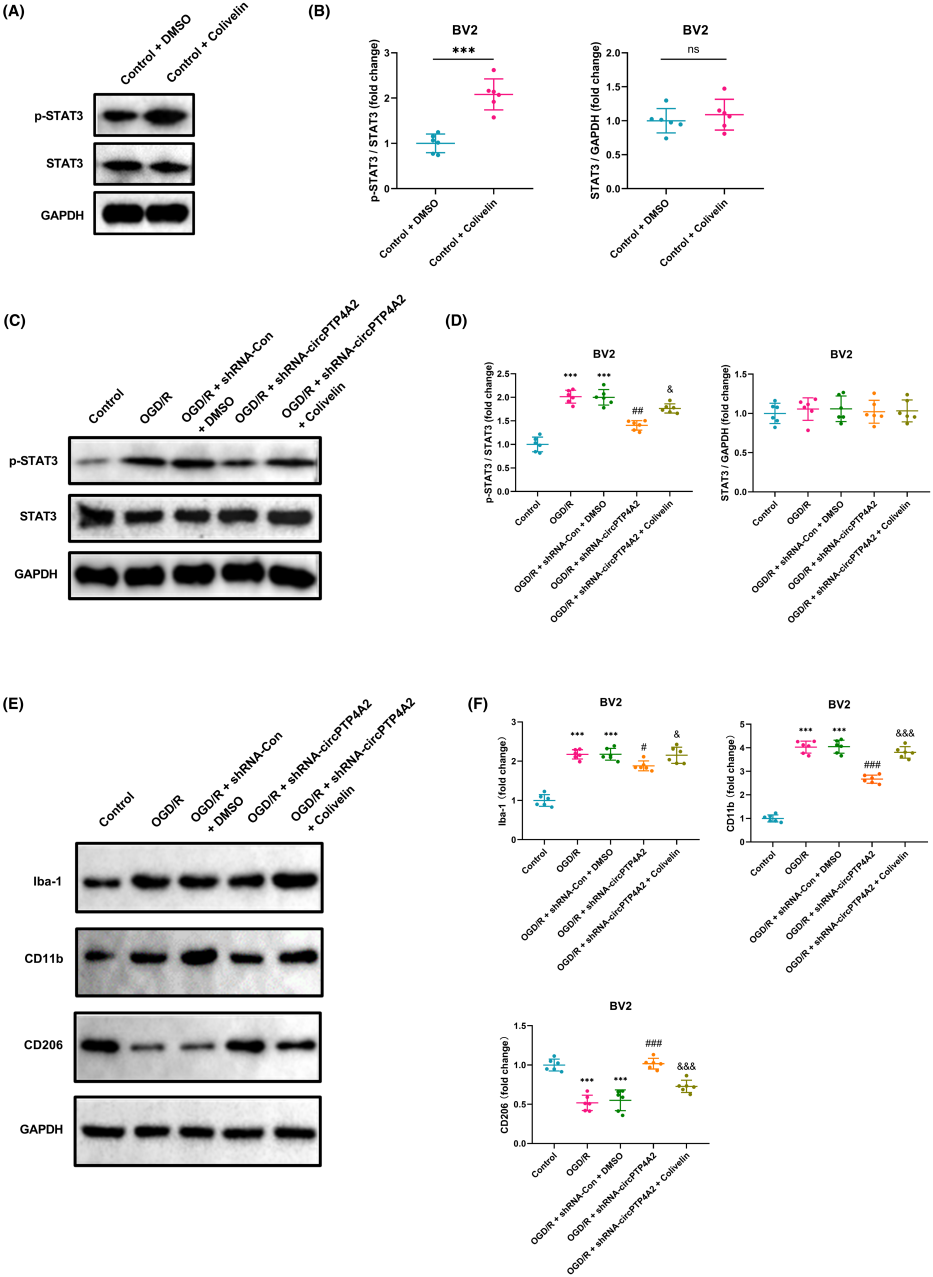
STAT3 activator (Colivelin) inhibits circPTP4A2 knockdown‐induced M1–M2 polarization of BV2 microglia under OGD/R condition. (A,B) Western blot analysis of p‐STAT3 and STAT3 in BV2 cells treated with Colivelin under normoxic conditions. *n* = 6. ***p < 0.001; ns, not significant. Student's *t* test. (C–F) BV2 were transfected with the circPTP4A2 shRNA lentiviruse and treated with Colivelin. Next, western blots were used to detect the expression of p‐STAT3; STAT3; Iba‐1; CD11b and CD206 in BV2 cells under OGD/R condition. *n* = 6. ***p < 0.001 versus Control group; ^#^ *p* < 0.05, ^##^ *p* < 0.01, ^###^ *p* < 0.001 versus OGD/R group; ^&^ *p* < 0.05, ^&&&^ *p* < 0.001 versus OGD/R + shRNA‐circPTP4A2 group. One‐way ANOVA followed by the Holm–Sidak test.

Previous studies have demonstrated that STAT3 is a crucial mediator of microglia polarization and inflammatory response, acting as a recognized regulator of inflammatory gene expression and a reliable indicator of ischemic stroke injury.^22^, ^23^ In response, we aimed to further investigate whether regulation of STAT3 through knockdown of circPTP4A2 could mediate the M1 to M2 polarization transition. As shown in Figure 5A,B, treatment with the STAT3 phosphorylation activator (Colivelin) effectively induced STAT3 phosphorylation in BV2 cells under normoxic conditions, while the whole levels of STAT3 remained unchanged. Moreover, we observed an increase in p‐STAT3 protein levels in BV2 microglial cultures following OGD/R induction (Figure 5C,D). After circPTP4A2 knockdown in BV2 cells exposed to OGD/R, we observed a significant decrease in p‐STAT3 expression compared to cells transduced with control shRNA lentivirus, while the overall levels of STAT3 protein remained constant. Furthermore, Colivelin‐induced upregulation of phosphorylated STAT3 partially rescued the reduction in p‐STAT3 levels caused by circPTP4A2 shRNA (Figure 5C,D). In addition, the Colivelin‐induced upregulation of phosphorylated STAT3 expression partially reversed the downregulation of Iba‐1 and CD11b levels, as well as the upregulation of CD206 expression, caused by circPTP4A2 knockdown in BV2 cells under OGD/R conditions (Figure 5E,F).

**FIGURE 6:**
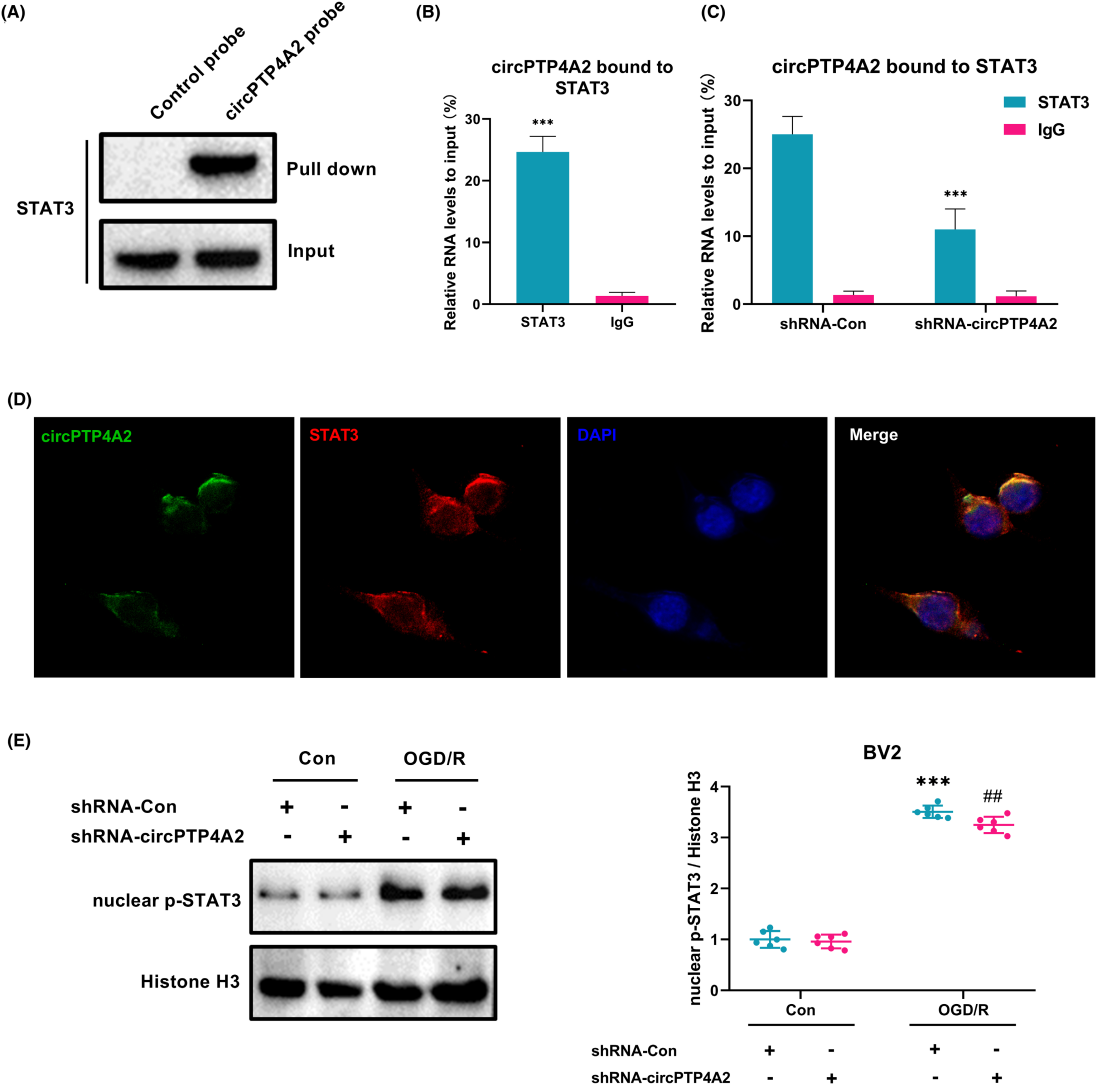
circPTP4A2 binds to the STAT3 protein. (A) Western blot of proteins pulled down by control and circPTP4A2 probes in BV2 microglial cultures following OGD/R induction using the STAT3 antibody. (B) RIP‐qPCR assay confirming the direct binding of STAT3 to circPTP4A2 in BV2 microglial cultures following OGD/R induction. ***p < 0.001 versus IgG. *n* = 6. (C) RIP‐qPCR assay detecting the specific binding of STAT3 and circPTP4A2 in BV2 microglial cultures following OGD/R induction by circPTP4A2 silencing. shRNA‐Con served as control. ***p < 0.001 versus shRNA‐Con group. *n* = 6. (D) FISH of circPTP4A2 (green), STAT3 (red), and DAPI (blue) in BV2 microglial cultures following OGD/R induction. (E) Western blot analysis of nuclear p‐STAT3 in BV2 cells. Cells were transduced with circPTP4A2 shRNA lentivirus for 48 h and treated with OGD for 3 h and reperfusion for 24 h. *n* = 6. ***p < 0.001 versus the control shRNA‐Con group. ^##^ *p* < 0.01 versus the OGD/R‐treated shRNA‐Con group.

According to the latest research, circRNA‐protein interactions influence protein expression and function, and are involved in diverse pathological and physiological processes.^24^, ^25^ To investigate whether circPTP4A2 could bind to STAT3, RNA pull‐down and RIP analyses were conducted in BV2 cells induced by OGD/R. The results of the Western blot analysis after RNA pull‐down showed that STAT3 were abundantly pulled down by circPTP4A2 probe compared with control probe in BV2 cells (Figure 6A). In addition, an RIP assay was carried out and circPTP4A2 was detected in the STAT3 immuno‐complex of BV2 cells (Figure 6B). The specificity of this interaction was confirmed by the decreased levels of STAT3 in BV2 cells transfected with shRNA lentivirus targeting circPTP4A2 (Figure 6C). Moreover, the immunofluorescence staining assay performed using a FAM‐labeled probe specific for circPTP4A2 and an anti‐STAT3 antibody demonstrated co‐localization of circPTP4A2 and STAT3 in BV2 cells (Figure 6D). We also measured nuclear p‐STAT3 protein levels in BV2 cells and found a significant increase in cells induced by OGD/R. However, transfection with circPTP4A2 shRNA lentivirus resulted in a significant reduction of nuclear p‐STAT3 protein (Figure 6E). Together, our results confirm an interaction between circPTP4A2 and STAT3, and suggest that the reduction of circPTP4A2 inhibits STAT3 phosphorylation in the nucleus of BV2 cells induced by OGD/R.

## 4 DISCUSSION

**FIGURE 7:**
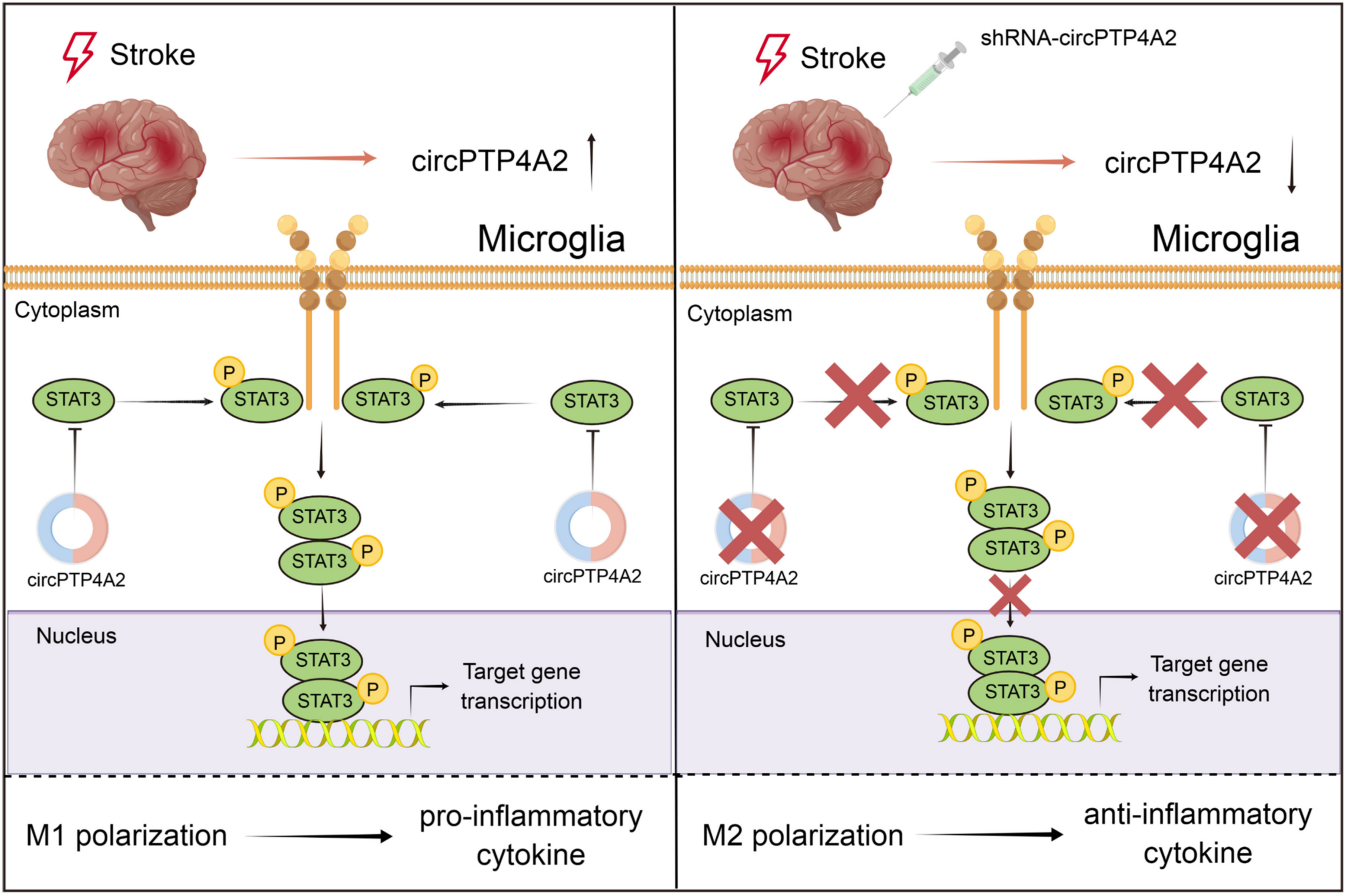
Schematic illustration of circPTP4A2 regulating microglial polarization in ischemic stroke. After cerebral ischemia, circPTP4A2 levels increased in peri‐infarct cortex and the elevation of circPTP4A2 was mainly located in microglia. Upregulated circPTP4A2 directly binds to STAT3 and promoted STAT3 phosphorylation activation. The phosphorylated STAT3 dimer then enters the nucleus to modulate stroke‐induced microglial polarization, which promotes neuroinflammation by driving M1 microglial polarization. Knockdown of circPTP4A2 using shRNA lentivirus results in neuroprotective effects via a mechanism that involves the downregulation of STAT3 phosphorylation and a subsequent shift in microglial polarization from M1 to M2 phenotype.

Our present study revealed a significant upregulation of circPTP4A2 expression in the ischemic tissues of the tMCAO mouse model. Knockdown of circPTP4A2 significantly reduced the infarct volume, increased cortical CBF, and alleviated neurological deficits during the early phase of stroke recovery. Moreover, circPTP4A2 knockdown in tMCAO mice downregulated proinflammatory factors while upregulating anti‐inflammatory factors, suggesting potential immunoregulatory mechanisms of circPTP4A2 after ischemic stroke. Remarkably, knockdown of circPTP4A2 in ischemic stroke promoted a phenotypic transformation of microglia from an M1 to an M2 state, potentially mediated by the downregulation of phosphorylated STAT3 (Figure 7). This study illuminates the role of circPTP4A2 in ischemic stroke, specifically its involvement in microglial polarization, which establishes a theoretical foundation for its potential application as a therapeutic target in the treatment of ischemic stroke.

Although our previous investigation revealed increased plasma levels of circPTP4A2 in ischemic stroke patients,^12^ the cellular source of this circRNA remains undetermined. In our mouse stroke model, we observed an upregulation of circPTP4A2 levels in both the plasma and peri‐infarct cortex of tMCAO mice. The increase in circPTP4A2 expression was more pronounced in the peri‐infarct cortex compared to the plasma. Importantly, the elevation of circPTP4A2 levels in the peri‐infarct cortex occurred prior to that observed in the plasma. However, we did not observe a significant increase in circPTP4A2 levels in WBCs of tMCAO mice, indicating that plasma circPTP4A2 may not have originated from WBCs. Moreover, our study has identified a positive correlation between plasma levels of circPTP4A2 and the peri‐infarct cortex, implying the disruption of the blood–brain barrier and subsequent release of circPTP4A2 from the brain into the peripheral blood. Hence, we postulate that the detection of circPTP4A2 in the plasma after ischemic stroke may partly originate from the brain. Further results revealed that knockdown of circPTP4A2 can enhance stroke recovery outcomes, implicating the involvement of circPTP4A2 in cerebral ischemic injury.

After the onset of ischemic stroke, neuroinflammation and immune responses are rapidly initiated within a few minutes, becoming the primary pathogenetic mechanisms within hours, which can persist for several months to several years.^26^, ^27^ Recent studies have demonstrated that circRNAs are involved in the pathogenesis of ischemic stroke by modulating immunoinflammatory reactions.^8^, ^9^, ^28^ Our previous bioinformatic analysis also suggested a potential association between circPTP4A2 and the immuno‐inflammatory mechanism of ischemic stroke.^12^ However, experimental verification is still required. Therefore, this present study continued to investigate the regulatory role of circPTP4A2 in neuroinflammatory injury during ischemic stroke. Our experimental results demonstrated that knockdown of circPTP4A2 effectively reversed the increase in pro‐inflammatory cytokines and decrease in anti‐inflammatory cytokines, indicating the involvement of circPTP4A2 in the initiation of neuroinflammation during ischemic stroke.

Microglia display dual roles in neuroinflammation, which are dependent on the microglial phenotype. Increasing evidence has suggested that shifting the microglial phenotype from a pro‐inflammatory M1 state to an anti‐inflammatory M2 state may represent an effective therapeutic strategy for ischemic stroke.^5^, ^6^, ^29^ Recent studies have demonstrated that Annexin A1 (ANXA1),^30^ Lipoxin A4 (LXA4)^31^ and recombinant nicotinamide phosphoribosyltransferase (NAMPT)^32^ exert neuroprotective effects by regulating microglial polarization. Consistent with these findings, knockdown of circPTP4A2 significantly reduced brain infarct volume, enhanced cortical CBF, and mitigated neurological deficits in tMCAO mice in vivo, while promoting M2 polarization of BV2 cells subjected to OGD/R in vitro. To our knowledge, this study is the first to demonstrate that downregulation of circPTP4A2 induces microglial switch toward an anti‐inflammatory M2 phenotype, revealing a novel role of circPTP4A2 in microglial polarization regulation. It is worth noting that, similar to the majority of studies in this field, our present study employed a binary M1/M2 polarization paradigm. However, the view that microglia are dichotomous and express solely M1 or M2‐specific markers has recently been disputed, and is considered an oversimplification.^33^, ^34^ Recently, the advent of single‐cell technologies and mass cytometry has facilitated the identification of novel microglial subtypes that exhibit complex molecular signatures.^35^, ^36^ Therefore, further studies are warranted to investigate the precise roles of circPTP4A2 in microglial polarization. While the crucial role of microglia as modulatory cells in physiological and pathological alterations of CBF is well established, the involvement of microglial polarization in CBF changes following ischemic stroke remains understudied.^37^ This suggests that factors other than microglial polarization may contribute to the observed decrease in CBF after circPTP4A2 knockdown. Notably, circPTP4A2 exhibits high expression in primary endothelial cells. Increased oxidative stress in these cells could potentially enhance the vascular endothelial permeability, disrupt cerebral vascular dynamics, and ultimately lead to decreased CBF.^38^ However, further investigation is required to ascertain the exact role of circPTP4A2 in mediating post‐ischemic CBF changes through endothelial cell modulation.

As a member of the STAT protein family of transcription factors, STAT3 is a well‐established regulator of inflammatory gene expression and is rapidly activated during the acute phase of cerebral ischemia.^39^ Accumulating evidence has demonstrated that STAT3 activation mediates pro‐inflammatory responses in microglia during ischemic stroke.^22^, ^23^, ^40^ Therefore, we investigated whether STAT3 is involved in the neuroinflammatory response of circPTP4A2. According to our experimental results, BV2 cells exposed to OGD/R showed increased expression of phosphorylated STAT3 (p‐STAT3), which was effectively suppressed by knockdown of circPTP4A2. In addition, treatment with a STAT3 phosphorylation activator reversed the circPTP4A2 knockdown‐induced phenotypic shift from M1 to M2 in BV2 cells exposed to OGD/R, thereby providing evidence that circPTP4A2 promotes M1 to M2 phenotypic conversion of microglia through inhibition of STAT3 phosphorylation. Currently, there is still controversy regarding the impact of STAT3 on microglial polarization during ischemic stroke. Wang et al.^41^ confirmed that Non‐erythropoietic mutant erythropoietin (MEPO) facilitated microglial polarization toward the protective M2 phenotype by promoting STAT3 activation. On the contrary, a recent study demonstrated that IL‐13 promoted the polarization of microglia toward the M2 phenotype by inhibiting STAT3 phosphorylation after ischemic stroke.^22^ The latter study is consistent with our findings, as we observed a shift in microglial phenotype toward an anti‐inflammatory state, providing further support for the beneficial effects of inhibiting STAT3 phosphorylation after ischemic stroke. Overall, our findings support the hypothesis that circPTP4A2 triggers microglial M1 polarization by activating STAT3 phosphorylation and that subsequent knocking down circPTP4A2 inhibits STAT3 phosphorylation and shifts microglial polarization from M1 to M2 phenotype, resulting in neuroprotective effects after ischemic stroke.

With the intensive research on circRNA in recent years, the pattern of circRNA‐protein interactions has received more attention.^24^, ^25^, ^42^ CircRNA‐protein interactions have been reported to influence protein expression, biogenesis, and pathophysiological processes.^42^ Previous research has confirmed that STAT3 can bind to circFAT1 as a circRNA‐binding protein, thereby activating the STAT3 pathway.^43^ Additionally, circRNAs have been shown to possess the remarkable ability to recruit STAT3 to specific cellular locations, as exemplified by Circ‐Amotl1, which is capable of recruiting STAT3 from the cytoplasm to the nucleus.^44^ Recently, there has been a report in the field of ischemic stroke research on the emerging mechanism of circRNA‐STAT3 interaction and its functional role. Specifically, circFOXP1 was found to regulate apoptotic signaling through binding to and modulating STAT3 activity in models of cerebral ischemia.^45^ This study demonstrated that circPTP4A2 binds to STAT3, and knockdown of circPTP4A2 inhibits the nuclear expression of STAT3 in BV2 microglia cells subjected to OGD/R. It is therefore plausible to speculate that circPTP4A2 can regulate microglial polarization by affecting the cytoplasmic/nuclear translocation of STAT3 and, thus, impacting neuroinflammation in ischemic stroke. In all, our findings provide a novel insight into the mechanism of circular RNA‐protein interactions in ischemic stroke.

Several limitations of our study should be acknowledged. Firstly, we partially used BV2 cells in our study, as they have been widely utilized in microglial research for their capacity to maintain primary microglial cell characteristics and functions.^46^ However, primary microglial cells are still considered the optimal choice for microglial studies. Secondly, our current study lacks data regarding the effects of circPTP4A2 overexpression on microglial polarization in both in vivo and in vitro settings. Thirdly, our analysis was limited to the impact of circPTP4A2 on the expression levels of cytosolic and nuclear STAT3 protein. Further investigation is warranted to elucidate the underlying molecular mechanisms by which circPTP4A2 modulates the cytoplasmic/nuclear translocation of STAT3 in microglia following ischemic stroke.

## 5 CONCLUSION

In summary, our findings demonstrated that circPTP4A2 plays a role in the promotion of brain injury and neuroinflammation following cerebral ischemia. Mechanistically, circPTP4A2 regulates microglial polarization in response to ischemic stroke by binding to STAT3 and modulating its phosphorylation activation, as revealed by our study. Our results provide supporting evidence for the physiological significance of circPTP4A2, laying a foundation for further exploration of its clinical potential as a therapeutic target.

## AUTHOR CONTRIBUTIONS

Xingzhi Wang, Zuohui Zhang, and Guiyun Cui designed the study. Xingzhi Wang, Shenyang Zhang, Bingchen Lv, Lei Bao, Miao Wang, Yan Wang, Wenqi Mao, Likun Cui, Ye Pang, and Fei Wang performed the experiments. Hao Chen, Wei Zhang and Liguo Dong provided help with data analyses. Xingzhi Wang drafted the manuscript. Guiyun Cui, Zuohui Zhang and Fuling Yan reviewed and provided advice for this manuscript. All authors read and approved the final manuscript.

## FUNDING INFORMATION

This study was supported by the Science and Technology Project of Xuzhou Health Commission (XWKYHT20220156), the Open Project of Key Laboratory of Colleges and Universities in Jiangsu Province (XZSYSKF2022021), the Science and Technology Planning Project of Xuzhou (KC19131, KC20113), Research and innovation program for graduate students in Jiangsu Province (KYCX22_2947, KYCX22_2948, KYCX23_2985), Leadership program through open competition in Xuzhou Medical University (JBGS202203), Key R&D Program (Social Development) of Jiangsu Province (BE2021630), Cerebrovascular Disease Youth Innovation Fund (Z‐2016‐20‐2201), and National Natural Science Foundation of China (82171305, 82301493).

## CONFLICT OF INTEREST STATEMENT

The authors declare that they have no competing interests.

## Supporting information


Data S1.

Data S2.

## Data Availability

The data supporting the findings of this study are available from the corresponding authors upon reasonable request.
