# Supplementary material for: Circular RNA PTP4A2 regulates microglial polarization through STAT3 to promote neuroinflammation in ischemic stroke
Source: CNS Neurosci Ther. 2023 Oct 23;30(4):e14512. doi: 10.1111/cns.14512 (PMC11017462; doi:10.1111/cns.14512)
Supplement: Supplementary file 1 — Data S1. [file CNS-30-e14512-s002.docx]

**Supplementary Information**

Table S1 Primers and RNA sequences for qPCR.

| **Name** |  | **Sequence** (5’-3’) |
| --- | --- | --- |
| circPTP4A2 (Div) | Forward | TGGTTCGAGTTTGTGATGCTACA |
|  | Reverse | CCAGAGTCGTCAAGGCAATGTT |
| circPTP4A2 (Con) | Forward | ACATTGCCTTGACGACTCTGG |
|  | Reverse | TGCAGATGGATACCTTCAGCTT |
| GAPDH (Div) | Forward | CCTCTGACTTCAACAGCGACAC |
|  | Reverse | CCATCACGCCACAGTTTCC |
| GAPDH (Con) | Forward | GAACGGGAAGCTCACTGG |
|  | Reverse | GCCTGCTTCACCACCTTCT |
| PTP4A2 | Forward | CCAATGCGACTCTCAACAAGTTCAC |
|  | Reverse | CAACACAACAGCCTGGCTCTTCAC |
| U6 | Forward | CGCTTCGGCAGCACATATAC |
|  | Reverse | TTCACGAATTTGCGTGTCATC |

Table S2 Biotinylated RNA probes used for RNA pulldown (5’-3’)

| **Name** | **Sequence** (5’-3’) |
| --- | --- |
| control probe | AAACTGTATGGCACAAAGGA- /3bio/ |
| circPTP4A2 probe | CGAAAAACTAGAACGTGGAT- /3bio/ |


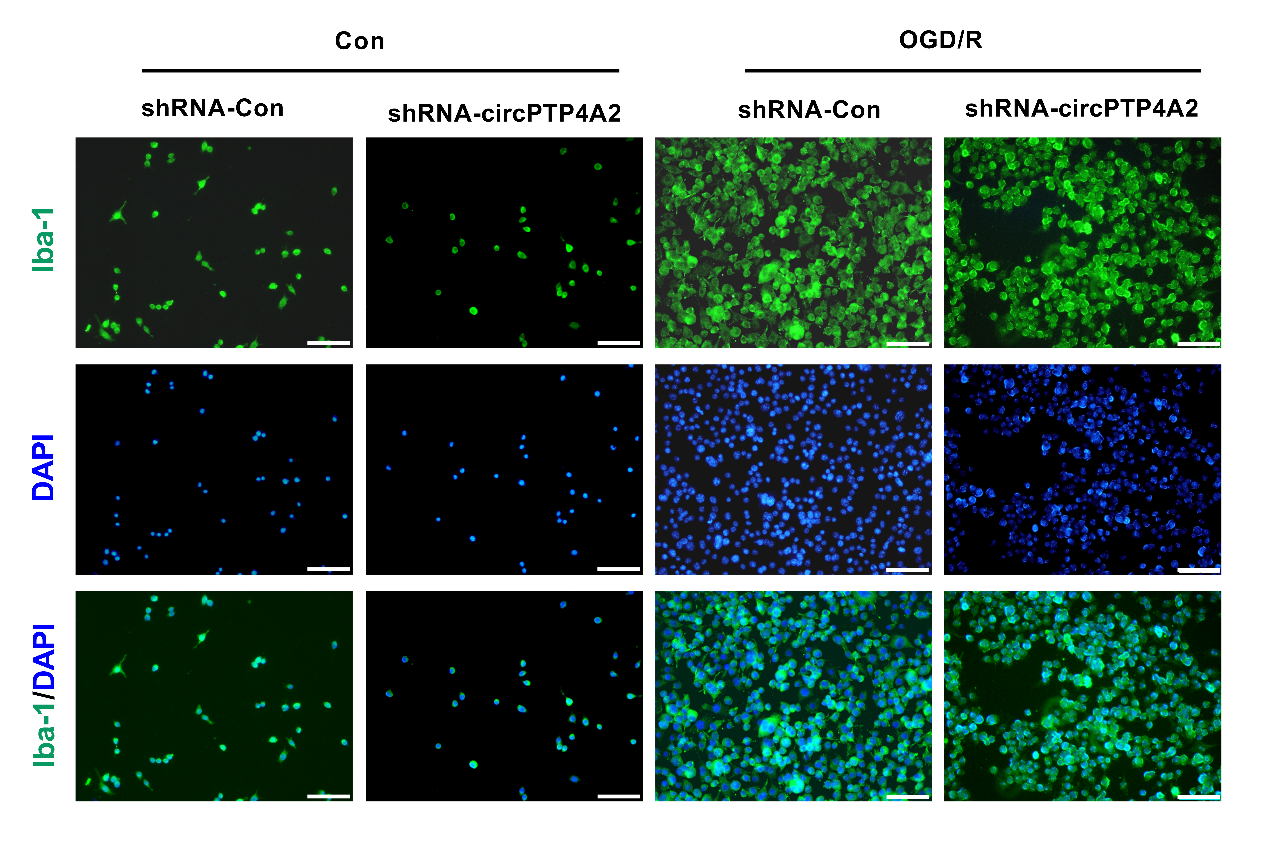


FIGURE S1 Immunofluorescence staining demonstrating the morphology of BV2 microglia in the resting and the activated state. Green, Iba-1; Blue, DAPI. Scale bar: 50 μm.
