# Supplementary material for: Circular RNA PTP4A2 regulates microglial polarization through STAT3 to promote neuroinflammation in ischemic stroke
Source: CNS Neurosci Ther. 2023 Oct 23;30(4):e14512. doi: 10.1111/cns.14512 (PMC11017462; doi:10.1111/cns.14512)
Supplement: Supplementary file 2 — Data S2. [file CNS-30-e14512-s001.pdf]

Full unedited gel/blot for FIGURE 1C

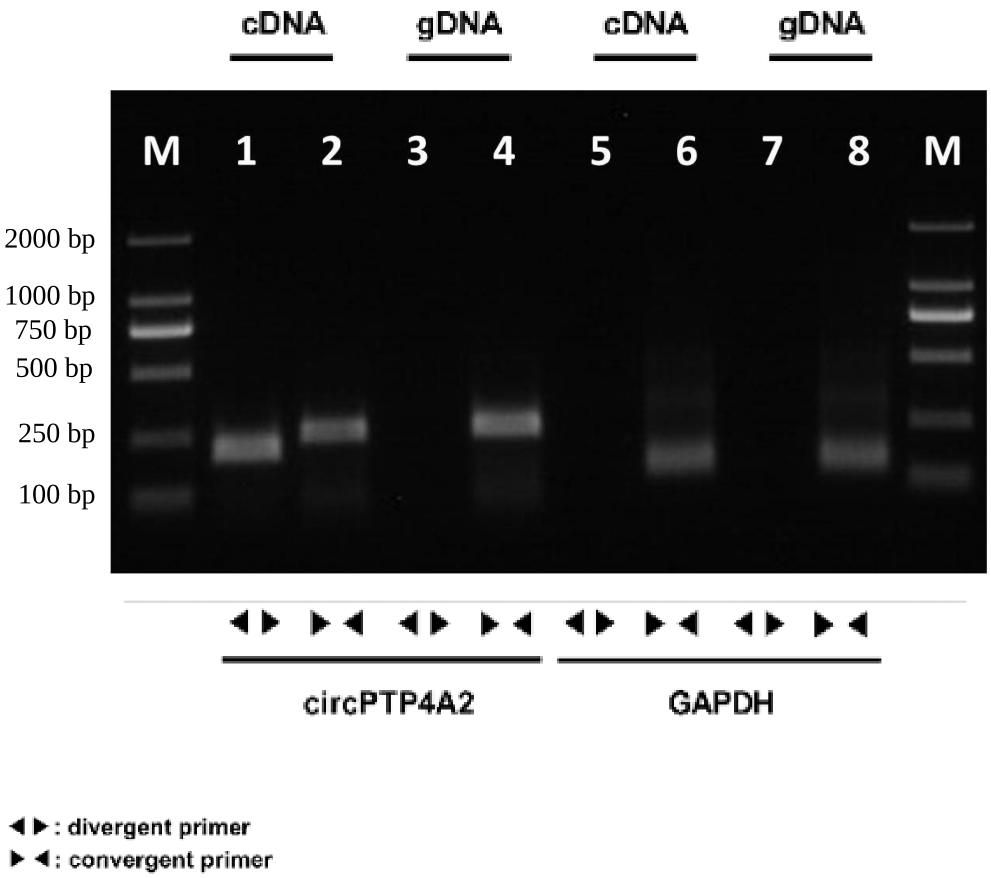

Full unedited gel/blot for FIGURE 3C

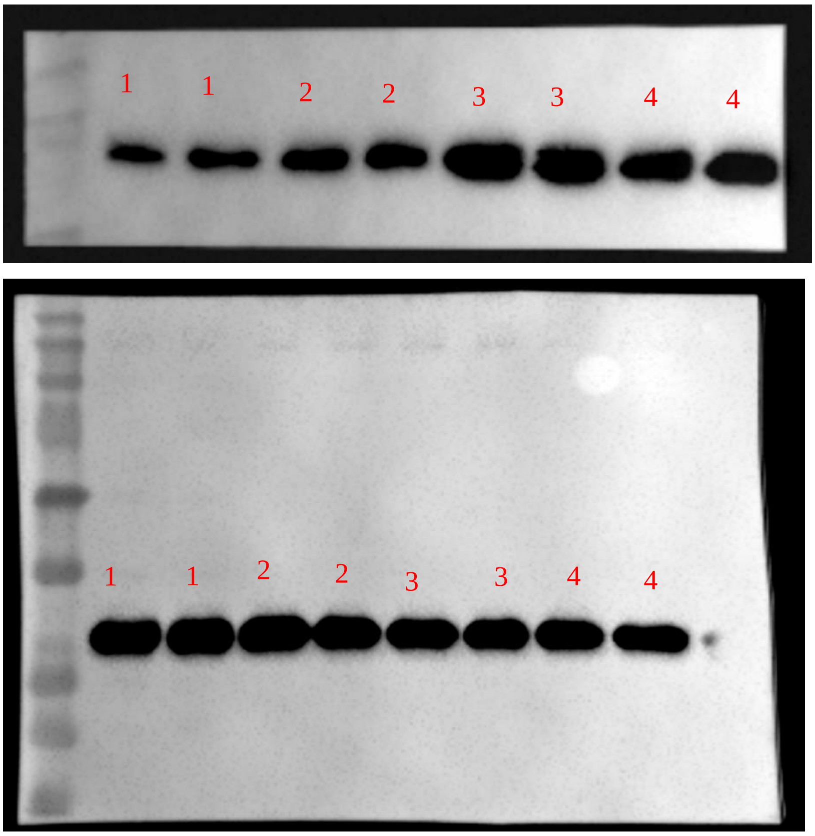

1: Sham + shRNA-Con
2: Sham + shRNA-circPTP4A2
3: tMCAO (R72h) + shRNA-Con
4: tMCAO (R72h) + shRNA- circPTP4A2

Full unedited gel/blot for FIGURE 3G

1: tMCAO (R72h) + shRNA-Con
2: tMCAO (R72h) + shRNA- circPTP4A2

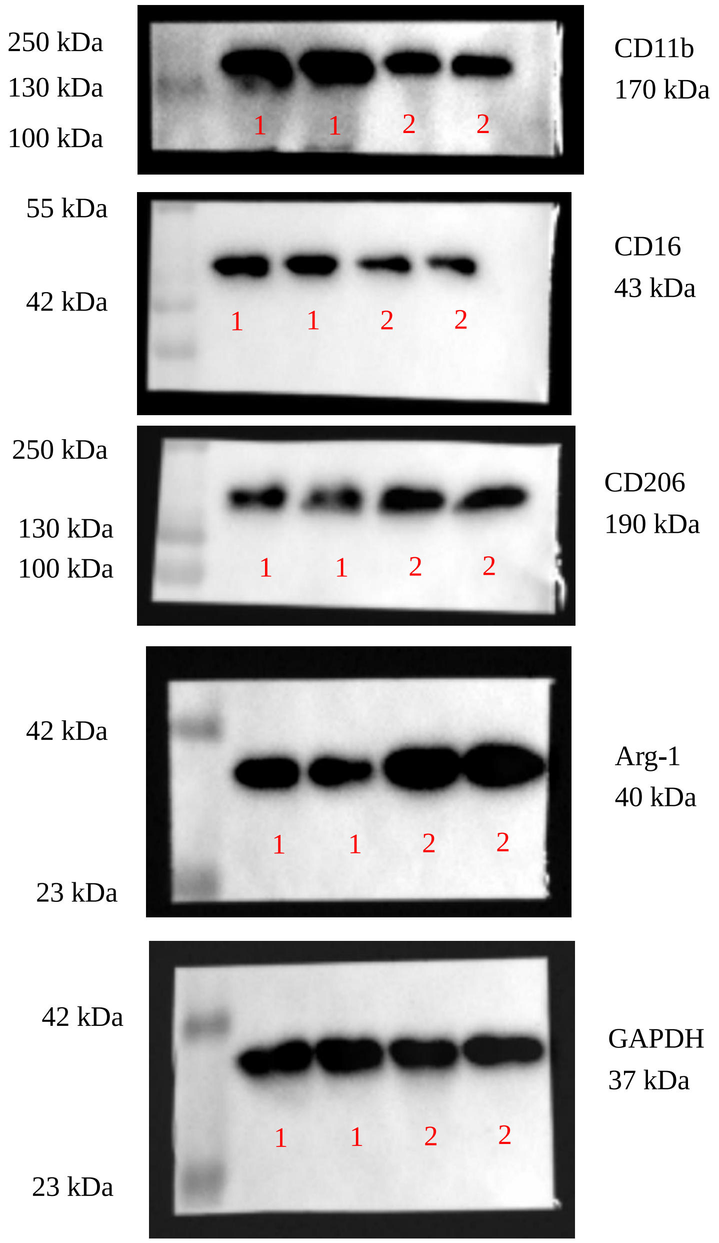

Full unedited gel/blot for FIGURE 4H

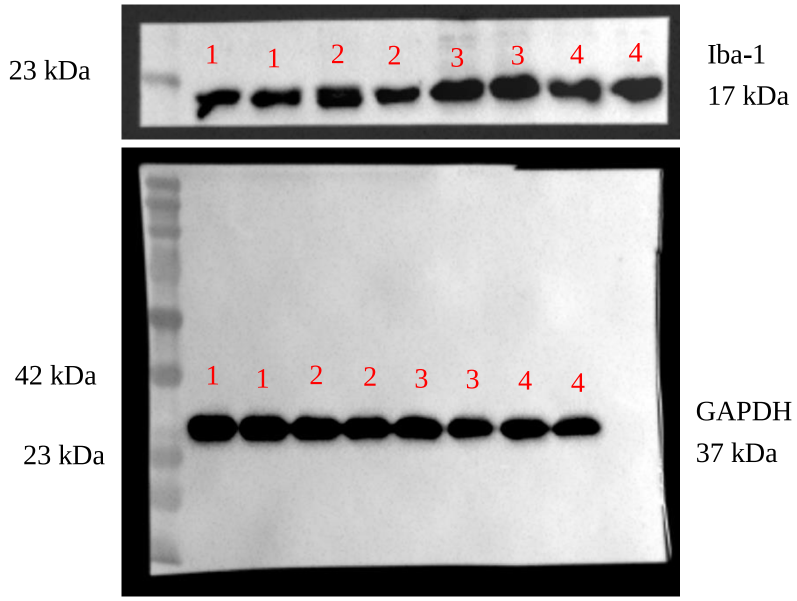

1: Con + shRNA-Con
2: Con + shRNA-circPTP4A2
3: OGD/R + shRNA-Con
4: OGD/R + shRNA-circPTP4A2

Full unedited gel/blot for FIGURE 4L

1: OGD/R + shRNA-Con
2: OGD/R + shRNA-circPTP4A2

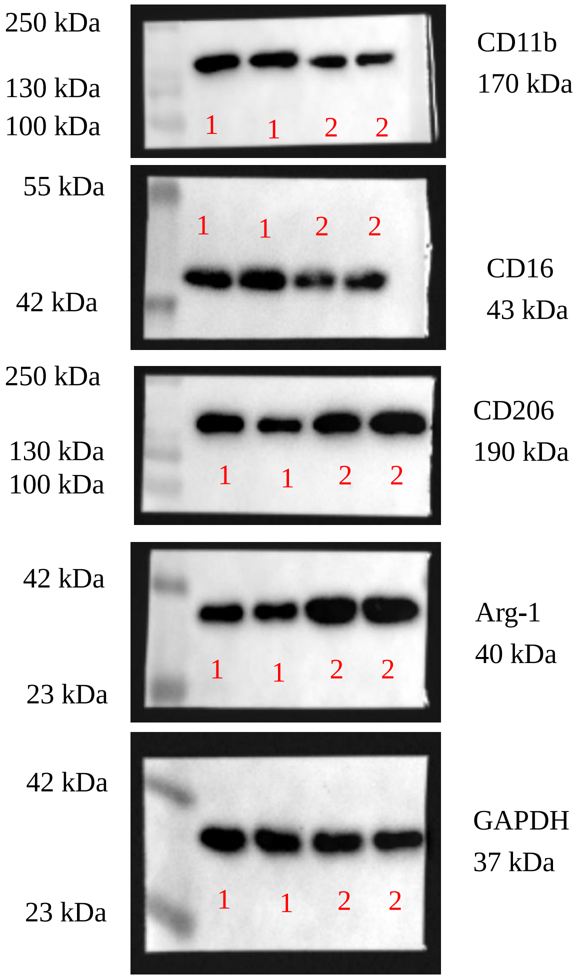

Full unedited gel/blot for FIGURE 5A

1: Control + DMSO
2: Control + Colivelin

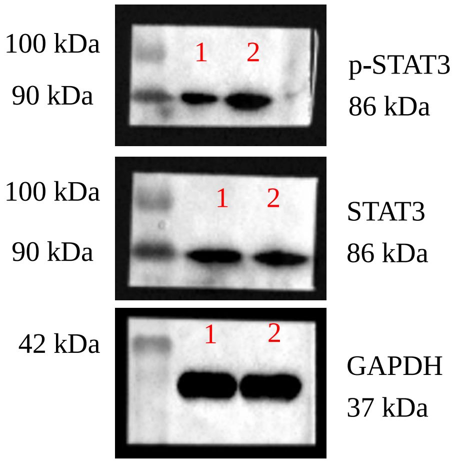

Full unedited gel/blot for FIGURE 5C

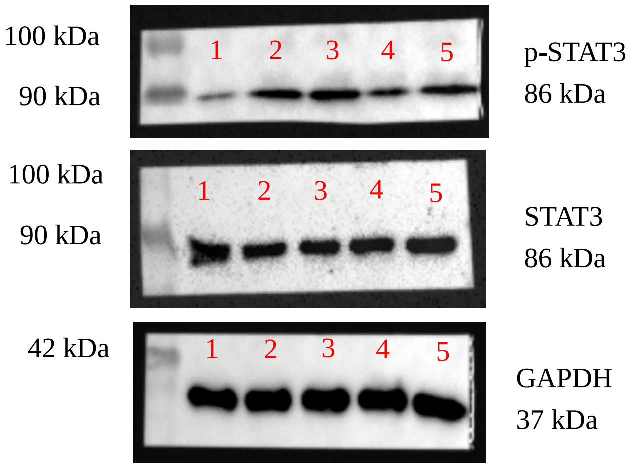

1: Control
2: OGD/R
3: OGD/R + shRNA-Con + DMSO
4: OGD/R + shRNA-circPTP4A2
5: OGD/R + shRNA-circPTP4A2 + Colivelin

Full unedited gel/blot for FIGURE 5E

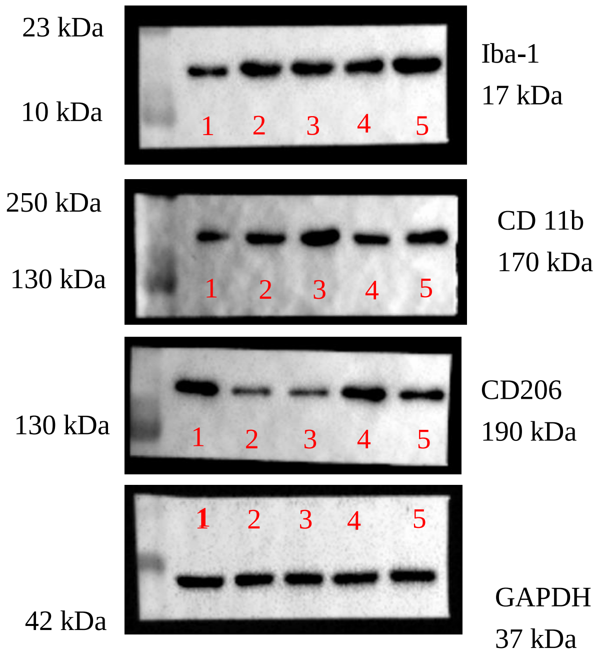

1: Control
2: OGD/R
3: OGD/R + shRNA-Con + DMSO
4: OGD/R + shRNA-circPTP4A2
5: OGD/R + shRNA-circPTP4A2 + Colivelin

Full unedited gel/blot for FIGURE 6A

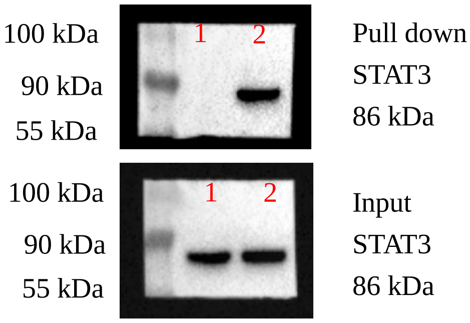

1: Control probe
2: circPTP4A2 probe

Full unedited gel/blot for FIGURE 6E

90 kDa
55 kDa
23 kDa

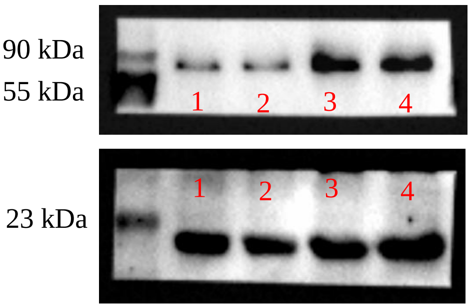

nuclear p-STAT3
86 kDa
Histone H3
17 kDa

1: Control + shRNA-Con
2: Control + shRNA-circPTP4A2
3: OGD/R + shRNA-Con
4: OGD/R + shRNA-circPTP4A2
